# Leveraging AI-enhanced digital health with consumer devices for scalable cardiovascular screening, prediction, and monitoring

**DOI:** 10.1038/s44325-025-00071-9

**Published:** 2025-07-02

**Authors:** Aline F. Pedroso, Rohan Khera

**Affiliations:** 1 Section of Cardiovascular Medicine, Department of Internal Medicine, Yale School of Medicine, New Haven, CT USA; 2 Cardiovascular Data Science (CarDS) Lab, Yale School of Medicine, New Haven, CT USA; 3 Center for Outcomes Research and Evaluation, Yale-New Haven Hospital, New Haven, CT USA; 4 Section of Health Informatics, Department of Biostatistics, Yale School of Public Health, New Haven, CT USA

**Keywords:** Cardiology, Health care

## Abstract

Traditional cardiovascular care relies on episodic, resource-intensive evaluations. Consumer wearable and portable devices, combined with artificial intelligence (AI), offer a scalable, low-cost alternative. These devices can enhance care with high-fidelity cardiovascular data captured outside traditional care settings, with AI further increasing their value. This review explores how AI-enhanced digital health tools can transform cardiovascular care, improving early detection, personalized risk assessment, and proactive management, particularly in resource-constrained settings, while bridging gaps in traditional care models.

## Introduction

Traditional cardiovascular care pathways have relied on episodic, resource-intensive evaluations led by expert clinicians in healthcare settings^1^. While advances in cardiovascular diagnostics have enabled improved detection and prognostication of disease in such care settings, this strategy has low accessibility and limited ability to scale to communities^2–4^. Such challenges are compounded in resource-constrained settings where routine screening and diagnosis are broadly limited by access to traditional preventive care^5^. However, AI-enabled digital health tools are set to transform cardiovascular care pathways through scalable, low-cost strategies that can improve the timely detection, prevention, and management of cardiovascular disorders directly in the communities^6,7^. Two synergistic developments have the potential to substantially alter the landscape of cardiovascular care.

First, a broad range of low-cost and highly scalable digital health tools now have sensors that enable high-fidelity and high-quality cardiovascular data to be captured directly in the communities on compact and mobile devices (portable) and devices that are worn by individuals (wearable)^8–11^. For instance, while previously electrocardiograms (ECG) and cardiac imaging were largely restricted to healthcare settings, ECGs can now be obtained on handheld and wrist-worn devices^12–14^. Similarly, the heart can be visualized by a point-of-care ultrasound (POCUS) plugged directly into a smartphone^15,16^. Therefore, these technologies have the potential to enable accurate, scalable, and accessible cardiovascular screening.

Second, while the acquisition of health data in the communities can broaden access, the reliance on the limited number of experts to interpret these studies restricts their potential scalability^17^. Recent advances in artificial intelligence (AI) can infer disease patterns from a range of cardiovascular diagnostic modalities^1,18^. However, most of these applications have been limited to testing in clinical settings. The development of AI tools specifically designed to expand inference from portable and wearable devices can bring a digital health-enabled revolution in community cardiovascular care^9,19^.

In this review we have addressed a broad range of novel methods, including machine learning, deep learning, and large language models (LLMs), along with their applications to signal and imaging processing. We explore the literature on the transformative potential of AI-enhanced digital health devices in cardiovascular screening, prediction, and monitoring in communities across care settings. The review contrasts traditional and AI-enabled care pathways and highlights how these advances can drive equitable access to preventive care, improve health outcomes, and reduce the global burden of cardiovascular diseases.

## Traditional approaches to cardiovascular care

Community-based cardiovascular care currently relies on reactive, episodic models of healthcare delivery, driven by healthcare encounters primarily initiated by the patients during scheduled routine visits or when symptoms appear^20^. In this section, we describe the current approach for screening, diagnosing, and monitoring these diseases in the community.

Currently, the screening for cardiovascular diseases primarily focuses on the collection of clinical and behavioral data to estimate the longer-term individual risk for major cardiovascular events^21–23^. Screening often includes a review of family history and assessment of risk factors to determine high-risk populations that may require advanced evaluations for a better assessment of an individual’s cardiovascular health^24,25^. Sporadic community-based screening programs with large-scale data collection complement these efforts and provide the opportunity to identify individuals at elevated risk who may benefit from early intervention^25^. While effective for identifying high-risk individuals and recommending lifestyle modifications, the current approach for cardiovascular screening often misses the opportunity to capture individuals in the early stages of disease or those with subclinical disease^26^.

After identifying those at risk, the actual diagnostic workflow depends on advanced laboratory and imaging studies to further evaluate cardiac structure and function^26^. Diagnosis is often guided by standardized clinical protocols that depend on collecting a series of healthcare parameters for a systematic evaluation of patient symptoms and risk factors^25,27^, which are particularly challenging in low-resource settings^28^. Additionally, conventional diagnosis strategies often depend on laboratory testing and expert interpretation of test results, which require infrastructure and skilled personnel, making them less accessible^29^.

In the context of cardiovascular disease monitoring, traditional models often heavily rely on the patient to track their symptoms, maintain the recommended changes in lifestyle, and comply with risk-reducing medications. It is also the individual’s responsibility to seek medical attention when necessary, with most patients limited by resources, an awareness of their health status, or even motivation to allow such an approach to be broadly successful^30,31^.

**Fig. 1:**
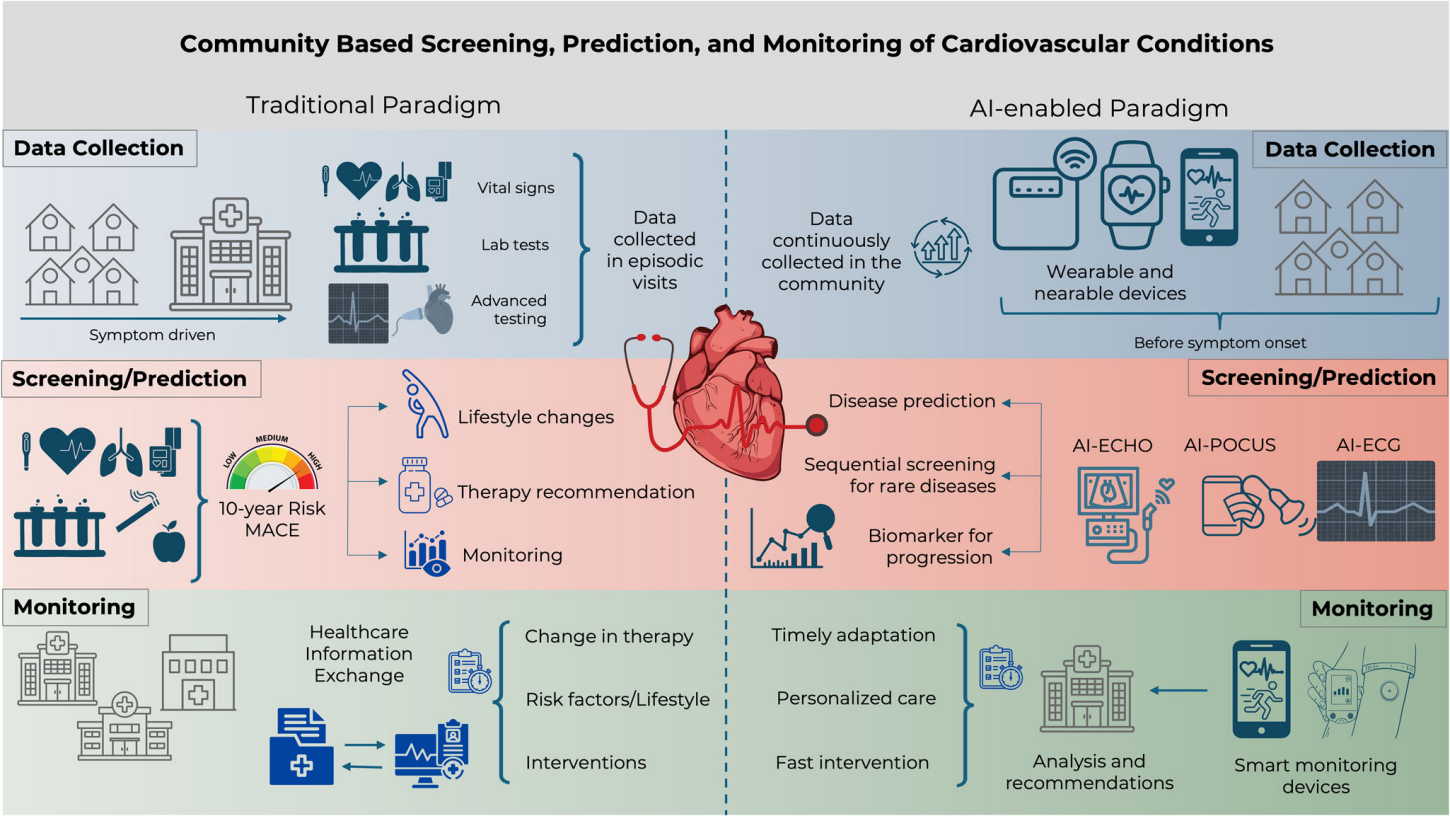
Traditional vs. artificial intelligence (AI)-enabled paradigms of cardiovascular care.

In the following sections, we present an overview of emerging data modalities being collected from consumer-facing devices and explore how AI can enable these data streams to advance cardiovascular care. Figure 1 contrasts the traditional and the AI-enabled paradigms of community cardiovascular care.

## AI-enhanced approaches to cardiovascular care

**Fig. 2:**
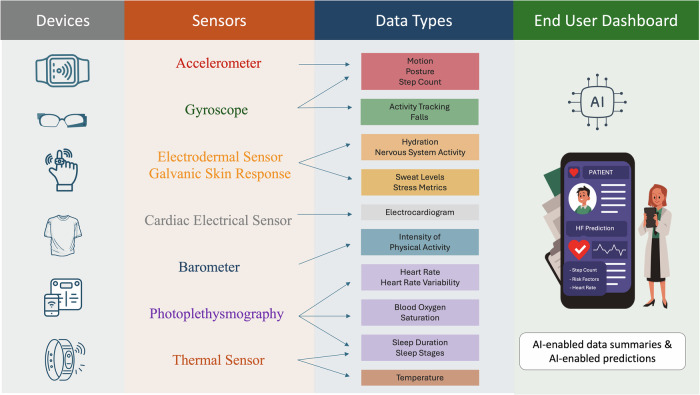
Emerging devices, sensors and data modalities being collected in the community by portable and wearable devices.

The rapid proliferation of wearable and portable health devices has led to an unprecedented increase in health-related data generated outside traditional healthcare settings^8,9^. Devices that are worn by individuals, such as smartwatches, rings, wristbands, shirts, and eyeglasses (wearables), and others that capture health information by being in the vicinity of individuals, such as smartphones, smart-scales, and others (nearables) (Fig. 2) can continuously collect physiological and behavioral data from individuals in the communities^32,33^.

### Emerging data sources from consumer devices

Advances in sensor technology integrated into several consumer-facing devices have enabled the collection of vast amounts of data with significant potential for applications in cardiovascular care^8,9^. These data sources are largely continuously collected without requiring user interaction, but there are also new actively collected data streams that provide targeted in-depth snapshots of health status at specific time points. A summary of the sensors available in consumer-facing devices is presented in Fig. 2^7,34^.

Even among the continuously collected data from these sensors, there is a vast range of data elements that capture different aspects of cardiovascular health. For example, in most smartwatches and fitness trackers, optical sensors using light-based technologies, like photoplethysmography (PPG), continuously measure blood volume changes, enabling the estimation of heart rate, heart rate variability, and blood oxygen saturation (SpO2)^35,36^. Accelerometers and gyroscopes detect motion, posture, and physical activity. They are widely used to monitor step count, activity and its intensity, and critical events, such as falls. Thermal sensors can monitor changes in skin temperature. Galvanic skin response sensors, also known as electrodermal activity sensors, measure changes in skin conductivity, which are influenced by sweat gland activity and, indirectly, by autonomic nervous system activity^37,38^. Pressure sensor-based ballistocardiography (BCG) can measure the mechanical activity of the heart by detecting the body’s subtle movements resulting from the cardiac ejection of blood^39,40^. In wearable devices, BCG sensors capture these micro-movements in wrist-worn devices^41^. Many devices also have a barometer that measures atmospheric pressure and altitude. In wearable devices, barometers can track changes in elevation and pressure, providing insights into the intensity of physical activity, such as climbing stairs or hiking, as well as environmental conditions like altitude and pressure^41,42^.

Consumer-facing devices are now increasingly equipped with other dedicated sensors for specific data streams that require active user engagement^43^. These sensors provide high-value snapshots of cardiovascular health at specific time points. For example, the cardiac electrical sensor, available on most advanced smartwatches, provides a clinical grade 1-lead ECG on the wrist^44,45^. Wearable cuffs and cuffless devices can also combine data from optical and mechanical sensors to estimate blood pressure on demand^32^. While episodic, these measurements provide valuable insights into the users’ health.

### Cardiovascular applications of new wearable-derived data

These new data modalities provide opportunities for many cardiovascular applications. For example, the combination of data from different sensors, like accelerometer data combined with PPG and temperature sensors, can provide estimates of sleep duration, stages, and quality^46,47^. Along with heart rate and SpO2 data extracted from PPG sensors, these devices can inform care for conditions like heart failure (HF) and sleep apnea^9,46^. BCG sensors can further enhance sleep monitoring by detecting subtle vibrations in the chest and abdomen caused by cardiac and respiratory activities. This enables continuous sleep monitoring while extracting a range of key physiological information to better assess sleep quality^39^. Irregular sleep patterns, identified through these data, have been associated with cardiovascular risk factors, including hypertension and a range of atrial arrhythmias^47^.

Accelerometer and gyroscope data enable monitoring patterns of physical activity, which provide an additional axis for cardiovascular health. Combined with barometer information on altitude and pressure, these can additionally provide insights into the intensity of physical activity as well as be useful in tracking physiological responses to changes in altitude^48^, symptoms such as chest pain or shortness of breath during exertion, and a decrease in exercise tolerance^11^. Thermal and electrodermal sensors contribute to understanding stress-induced cardiovascular responses, such as transient elevations in heart rate and blood pressure, by providing data on stress-related autonomic nervous system activation^49,50^.

These applications broaden the more commonly recognized cardiovascular applications of dedicated cardiac sensors available on wearable devices. The most prominent example is the single-lead ECGs that identify irregular heart rhythms like atrial fibrillation (AF)^13,51^. These wearable-collected ECGs have been shown to facilitate long-term monitoring of cardiac rhythms outside clinical settings, capturing transient or asymptomatic events that might be missed during brief in-office assessments. AI applications to these data allow for screening and diagnosis of various cardiovascular conditions, including AF, stroke, cardiac arrest, HF, and even subclinical cardiomyopathies^52–54^, which are discussed in further detail in the following sections. Beyond traditional wearable devices, wearable cuff-based and cuffless devices that measure blood pressure and emerging optical sensor technologies that measure blood glucose non-invasively show promise in managing major cardiovascular risk factors^55,56^.

While these sensors generate high-quality health-related data, AI can further enhance their value and can enable a new frontier of cardiovascular care in the community. Combining diverse sensor data with AI can allow early detection of several unique cardiovascular risk markers, facilitate personalization of care, and further allow low-touch monitoring directly in the communities. Some recent clinical trials have explored how AI-enabled wearables can support therapy monitoring and clinical decision-making in real-world populations^57–59^. For example, RATE-AF was a randomized clinical trial (RCT) that used a consumer-grade wrist-worn wearable device in older adults with AF and HF to monitor over 140 million heart rate data points and over 23 million physical activity data points over a 20-week period. By processing the wearable-derived data with a deep learning model, the authors demonstrated that the functional status (defined by the New York Heart Association class) could be predicted with a performance equivalent to standard clinical assessments such as ECG and the 6-min walk test^60^. In another RCT conducted under both in-clinic and community conditions with patients with type 2 diabetes, an automated insulin delivery system enhanced by an AI-based predictive control algorithm that continuously integrated heart rate and accelerometry data from a smartwatch, reduced glucose variability and minimized hypoglycemic events without requiring user input during physical activity. The findings underscore how consumer-grade wearable devices, coupled with AI, can support personalized and adaptive care delivery beyond traditional healthcare settings^59^.

## AI-enabled utilization of wearable/portable device data in cardiovascular care

While new health data that can be acquired on wearable and portable devices offers novel avenues for cardiovascular care, achieving the full potential of these innovations requires their effective integration into healthcare systems^61,62^. AI applications can address challenges with managing the volume and quality of these data by enabling their optimization into usable metrics, filling gaps in incomplete datasets, and identifying critical patterns within these data^62^. The sections below explore specific AI use cases, including strategies for enabling data interoperability and integration into clinical workflows.

### AI-assisted interoperability of wearables with EHR

Interoperability refers to the ability of different systems, devices, and software to communicate and exchange information. In the context of wearable devices, diverse systems need to exchange and use the data available from these devices effectively^63,64^. A key barrier to interoperability in these data is the challenge of achieving both syntactic and semantic compatibility^65^. Syntactic interoperability ensures that data can be exchanged in standardized formats, while semantic interoperability ensures that the meaning of the data is preserved across different systems. Creating standardized approaches for these two key components ensures consistency in how data are captured, stored, and interpreted^57^. The growing number of proprietary wearable devices in clinical and consumer settings has introduced considerable variation in how physiological signals are captured and reported. A major challenge lies in the inconsistency of measurement definitions and the lack of calibration standards across manufacturers, which limits the interoperability of data. Without a uniform approach to how metrics like heart rate or activity are measured, comparing outputs across platforms or integrating them into unified datasets remains problematic. Differences in the timing and frequency of data collection pose an additional barrier. Devices may sample at irregular intervals or use context-dependent triggers, making temporal alignment across sources difficult, particularly in longitudinal monitoring or real-time clinical decision-making. The American Heart Association (AHA) and other professional organizations have called for coordinated efforts to develop validation frameworks and common data standards that can serve as the basis for scalable clinical integration. In the absence of achieving standardization at source, there is a potential role for AI to simplify the complex task of integrating data from multiple sources by identifying relationships between disparate datasets and merging them into a unified, consistent format^57^.

Semantic compatibility ensures that the meaning of data is preserved and understood uniformly across systems^66^. This concept is pivotal in enabling seamless integration of wearable device data into clinical workflows. For example, semantic interoperability requires the inference from these data to be mapped to clinically meaningful categories, which could be further standardized via vocabularies and terminologies to ensure that data captured by wearables can be interpreted consistently, regardless of the system or application using the data^67,68^. Achieving semantic compatibility involves not only aligning terminologies but also structuring data so that its clinical relevance is preserved during transmission and use^61^.

Concrete strategies to support syntactic and semantic interoperability include the adoption of standardized data formats such as HL7 Fast Healthcare Interoperability Resources (FHIR), which provide structured frameworks for exchanging health data across diverse systems^69^. Moreover, other innovations in natural language processing, especially the emergence of large language models, can enable the alignment of ontologies and can help map heterogeneous data elements into unified, clinically meaningful representations^70^.

### AI-assisted integration of wearable data into clinical workflows

Integrating wearable-derived data into clinical workflows is essential for their effective application in healthcare^71,72^. Clinicians across the world operate under significant constraints on their time, and the addition of new data streams risks overwhelming them with additional tasks, reducing efficiency, and potentially diverting attention from direct patient care. To be clinically valuable, wearable-derived data must be seamlessly incorporated into existing workflows without introducing new steps or requiring additional effort^73^.

**Fig. 3:**
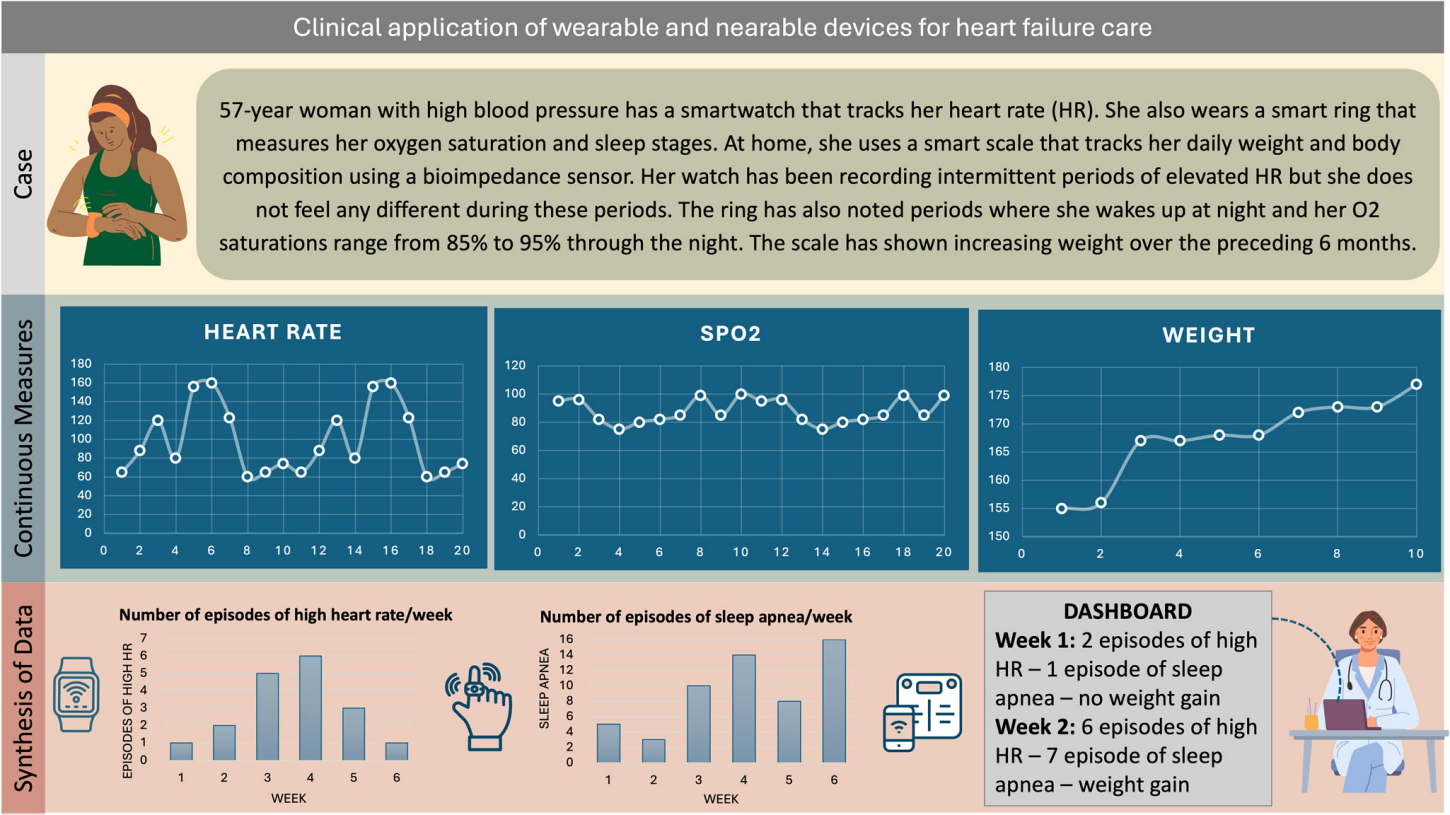
Clinical application in patients with heart failure.

Integrating these new data streams into existing workflows requires first synthesizing these large-volume, continuously collected data into interpretable summaries^74^. AI-driven data summarization can occur at two levels: within the healthcare system or through edge computing directly on the device^75^. Edge computing enables data analysis to be performed locally, reducing the need to transmit sensitive health information outside encrypted devices. This approach enhances data privacy, while also enabling more immediate feedback for critical scenarios^76^. By alleviating network congestion and reducing latency, edge computing also improves the responsiveness and reliability of AI-driven healthcare systems. Novel AI tools can streamline data integration into clinical workflows by automating repetitive tasks, such as data entry and report generation^64^. For example, AI tools can be applied to create clinically meaningful synthesis across multiple metrics^77^. A clinical scenario that conceptualizes such a setting is presented in Fig. 3. Meaningful integration must go beyond technical capability. To avoid overwhelming clinicians, AI systems should incorporate prioritization algorithms that triage information and surface only the most clinically relevant findings. Additionally, clinician-centered dashboards designed with input from end users can help translate raw data into intuitive, actionable insights that fit into existing decision-making processes.

Despite promising technological advances, several real-world considerations can limit the implementation of AI-enabled wearable solutions to improve cardiovascular care. A major challenge is the sustained use of these devices by patients, especially those that require daily engagement or manual data input^78^. Moreover, there are financial considerations that would limit adoption. Reimbursement pathways for both devices and AI algorithms are still evolving and often lack clarity, particularly in settings outside traditional fee-for-service models^8^. Additionally, integrating continuous data streams into clinical workflows can risk overwhelming providers unless thoughtfully managed through prioritization algorithms and an actionable interface. To support adoption, user-centered design principles rooted in human-factors engineering are essential. Wearable devices must be comfortable, unobtrusive, and intuitive while minimizing cognitive load for both users and clinicians^79^.

## Considerations for deploying AI-enabled devices in cardiovascular care

**Fig. 4:**
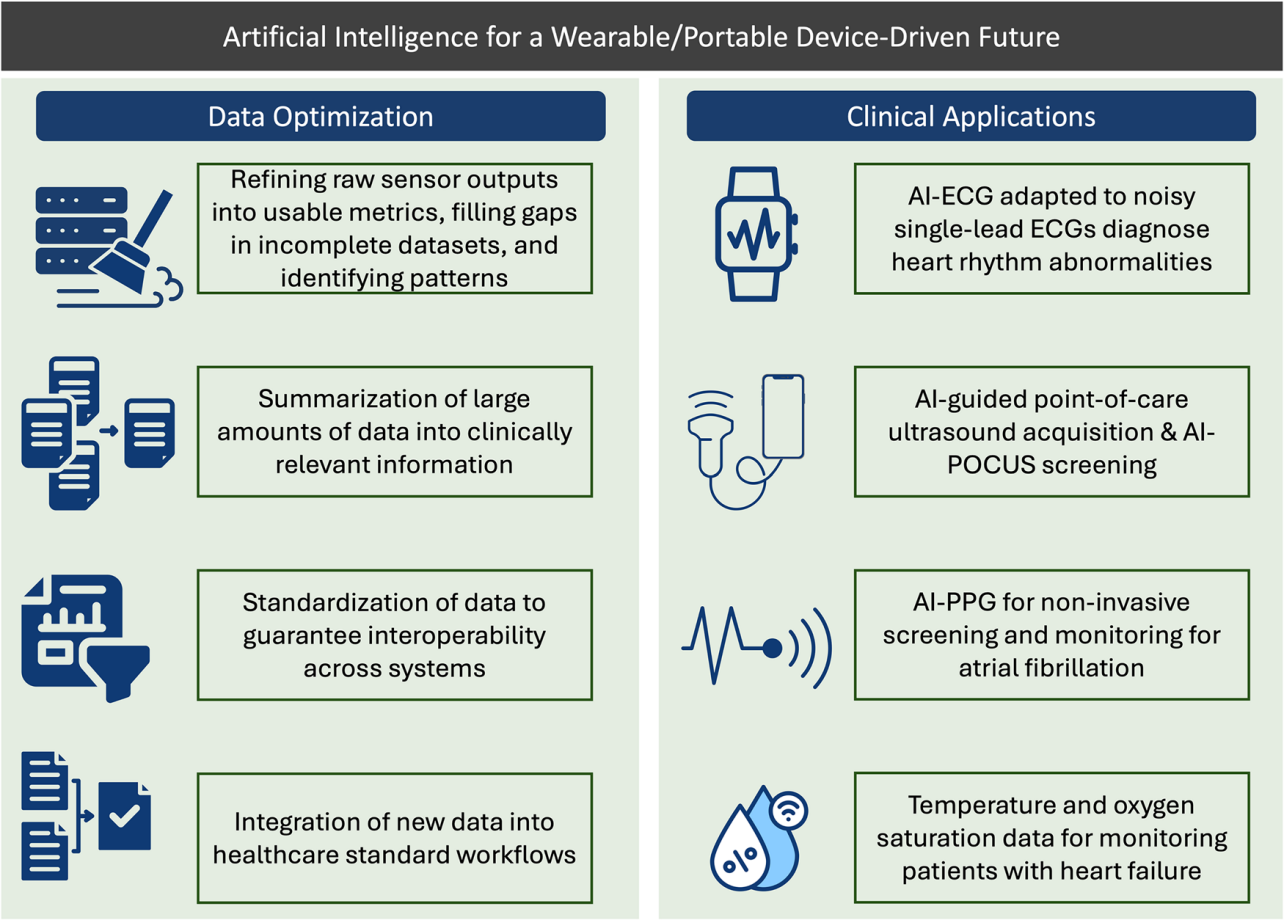
Artificial intelligence for a wearable and portable device-driven future.

Advances in AI have recently been applied to data generated by community health devices to improve many facets of cardiovascular care^1,10^. However, the translation of their outputs into clinical practice requires addressing a number of foundational challenges. In this section, we examine the key technical, clinical, and regulatory considerations involved in the development and deployment of AI-enabled cardiovascular solutions (Fig. 4). We discuss the key considerations for developing and leveraging these technologies in care, the major cardiovascular AI applications for these devices on the horizon, and provide a framework for novel care pathways that leverage these tools effectively.

### Technical considerations for developing clinical AI solutions for wearable devices

The development of AI for these digital health devices requires addressing six critical challenges.

#### Lack of large wearable repositories for model development

While 1.1 billion individuals reportedly use a wearable device worldwide^80^, generating 28 petabytes of data each year^81^, the data are largely fragmented and unavailable for disease discovery and optimizing care. Only a few studies have incorporated wearable device data into their frameworks. Among large discovery cohort studies, the UK Biobank, has accelerometer data from approximately 50,000 participants^82,83^. These represented two weeks of data from participants wearing these devices relatively continuously. However, this differs from the real world, where data collection spans more extended periods but with lower day-to-day use of the devices. Hence, the available data consist of more extended periods of sparse data, posing challenges for direct translation of inference on healthcare outcomes. Another cohort, the All of Us Research Program (AoURP) includes wearable device data from 13,000 participants, all sourced from a single device type (Fitbit)^84^. AoURP wearable data are not provided in a granular format but are instead aggregated into summary metrics such as daily step counts and sleep estimates. While the data have been used for examining outcome associations of wearable-derived metrics^47,85^, the lack of detailed, continuous data limits the potential for in-depth analysis and algorithm development.

#### Clinical correlates for wearable device data are often limited

A related but independent consideration for the development of AI from wearable devices is how well device-derived data are paired with validated markers of cardiovascular disorders and associated clinical outcomes. The distinct consideration for this element is essential because establishing participant-driven networks to create large repositories of wearable data is useful only if these data can be paired with corresponding clinical information for these patients. This contrasts the AI and cardiovascular innovations developed directly for healthcare data streams, where the vast available clinical correlates data have spurred across several domains^1,7,86,87^. Therefore, while data in health system EHRs contain the requisite clinical information on a vast number of patients, the lack of integration of data from their patients who use wearable devices precludes the effective use of these data. Certain emerging studies are addressing these challenges by systematically collecting wearable data^88,89^.

#### Quality of available data relative to traditional clinical data streams

The quality of data derived from wearable and portable devices differs significantly from traditional clinical data streams. These differences, influenced by the type of device, patterns of use, and quality of acquisition, have important implications for using such device-derived data in clinical care. Consumer devices vary in the accuracy of their sensors, how data are structured, and how they are outputted to the user. In that context, data sparsity is a common challenge arising from a combination of users inconsistently wearing these devices and their disengagement from continued use. For example, users may wear fitness trackers sporadically or remove smartwatches for swimming, charging, or sleeping, leading to gaps in continuous monitoring. In addition to data sparsity, wearable data are often affected by unwanted or irrelevant variations captured by the sensors that interfere with the accurate measurement of the intended signal^90^. Wearable ECG data is particularly prone to noise, resulting from poor electrode-skin contact, movement artifacts, muscle contractions during recording, and external electrical interference^91,92^. The challenges of data quality extend to other important handheld devices for community cardiovascular diagnosis, such as POCUS. While these devices broaden access to cardiac imaging, the quality of data they acquire is determined by the skill and experience of operators acquiring these images. Therefore, those without adequate training may acquire images of low quality, introducing variability to the data. However, many of the gaps in data quality can be directly addressed by certain key safeguards, such as broader engagement with patients and encouraging optimal use, especially for those whom the wearable and portable devices are used for health monitoring^93,94^. Moreover, for ECG and POCUS acquisitions, novel AI innovations, described in the following section, are designed to improve both the quality of acquisition and the reliability of inference from these noisy data.

#### Epidemiological considerations for deployment in real-world settings

The deployment of AI-based diagnostic tools in real-world settings must account for the challenges posed by the low prevalence of diseases among healthier populations in the community^95–97^. In such scenarios, even fairly accurate models can yield a significant number of false positive results, potentially leading to unnecessary additional testing that diminishes their clinical utility, especially when used in low-resource settings^98^. For example, a hypothetical model with 95% sensitivity and 90% specificity is applied to a population with disease prevalences of 1%, 5%, and 10%. At 1% prevalence, fewer than 1 in 10 individuals identified as positive by the model actually have the disease, but with prevalences of 5% and 10%, this represents a third and half of the tested individuals, respectively. This example highlights how the clinical value of a model is context-dependent: in high-prevalence or diagnostic settings, PPV improves, and sensitivity may take precedence to avoid missed diagnoses, whereas in low-prevalence, population-level screening, NPV is high, but maximizing specificity becomes important to reduce false positives and unnecessary follow-up. This issue underscores the need for AI models with tailored thresholds that optimize both sensitivity and specificity. In public health programs where resource constraints are present, high specificity may be prioritized to limit false positives and downstream testing burden, while in diagnostic settings, particularly when the cost of missed disease is high, greater sensitivity may be preferred. Moreover, there is a role for sequentially deployed low-cost screening strategies to minimize false positives in low-prevalence settings^99^. One effective approach could involve integrating multimodal data for case enrichment and implementing sequential screening to detect a range of cardiac abnormalities using AI tools^100^. This structured approach not only reduces the impact of false positives but also prevents unnecessary strain on healthcare systems.

#### Integration into healthcare workflows

Wearable and portable health devices often operate within proprietary ecosystems, which can limit interoperability. Tools from different manufacturers typically use distinct software to process and analyze data, thereby creating data silos that are challenging to aggregate into a unified platform. This lack of interoperability limits the linkage of AI inferences across devices. For instance, an AI model trained to analyze ECG data from one wearable device may not be compatible with data from another device, even if the underlying physiological information is similar. Application Programming Interfaces (APIs) are pivotal in enabling communication across devices, and their integration into healthcare data ecosystems. Moreover, they allow the deployment of AI models across devices by allowing sandboxes that aggregate data across devices. While API-based data access does not address all interoperability issues, it enables interfaces that facilitate real-time data exchange and inference that can be deployed across devices. Such an approach also allows healthcare organizations to integrate new technologies without significant system changes.

#### Regulatory and ethical strategies before deployment

The deployment of AI-powered diagnostic tools in healthcare requires consideration of evolving regulatory and ethical elements. The key questions include the reliability of these systems, their transparency and fairness, and the implemented processes to align with their actual use and how they are designed. Regulators typically consider wearable devices and consumer health technologies under their medical device framework. These regulations aim to ensure that devices marketed with claims of health monitoring or diagnostics meet specific safety and efficacy standards. Wearable devices that provide general wellness information may not fall under the same rigorous oversight unless intended for clinical use. Additionally, the FDA evaluates devices on their risk classification, considering factors such as the intended use and potential impact on patient safety. As AI-enabled wearables evolve toward continuous learning systems, regulatory frameworks must also adapt to this emerging framework. The FDA’s Software as a Medical Device (SaMD) guidelines, including its proposed Predetermined Change Control Plan, represent an essential step in allowing adaptive algorithms while ensuring safety and accountability. Another critical consideration is ensuring the appropriate use of the data generated by these new technologies. For instance, repurposing ECG outputs from consumer devices for off-label metrics beyond their approved indication may constitute a new intended use, which can subject the application to regulatory scrutiny under existing FDA pathways^101^.

These regulatory requirements set the stage for addressing ethical considerations such as ensuring patient autonomy, safeguarding data privacy, and promoting equitable use of AI-driven health applications. Variability in sensor performance across skin tones and disparities in digital literacy pose risks to equity and trust^102^. A critical question is whether these novel insights from patients’ devices should be shared directly with them so they can process and seek care based on their preferences. The alternative is that when information is derived from these emerging sources, which have built-in uncertainty, these are shared with clinicians who can ensure that these inferences are placed in the context of the overall clinical presentation, minimizing the risk of misinterpretation of information and psychological distress. This can be further compounded by the false positive results, which can cause distress without any potential beneficial effect for patients. However, to ensure the autonomy of the patients can be balanced with the safety of the application, the regulation of these health applications should be closely tied to their strategy for clinical use. Incorporating guidance from the World Health Organization (WHO) AI ethics and governance and other regional regulations can help mitigate these challenges through principles of transparency, inclusiveness, and algorithmic accountability^103^.

### AI for enabling cardiovascular health insights from wearable and portable devices

The potential of deep learning to derive clinical insights from wearable and portable devices has manifested in a range of early applications. There are 3 domains that have observed the most sustained development.

#### AI applications for PPG-sensor-derived data

A range of statistical, machine learning, and deep learning algorithms have enabled valuable insights from beat-to-beat data acquired by PPG sensors. Early applications used combinations of input features like inter-beat intervals and heart rate variability in linear models to examine association with a series of cardiovascular diseases, offering interpretable insights from these data^104^. More advanced machine learning models have enhanced the inference from these data by detecting complex features and their interactions, with more robust assessment of disease associations. More recently, deep learning models, such as those leveraging convolutional neural networks, long short-term memory networks and transformer models, have provided further automation in parsing wearable data and identifying hierarchical representations of PPG data and their disease associations^105,106^.

For example, a model using heart rate data continuously collected with PPG sensors was useful for AF monitoring^107^. Another investigation used deep learning methods on raw PPG waveforms to distinguish AF from sinus rhythm. This approach, validated in patients undergoing cardioversion, outperformed conventional heart rate variability metrics and models based on heart rate alone^108^. The study underscores PPG’s utility in AF management, not only for screening large populations but also for tracking AF episodes and guiding rate control. While challenges like signal artifacts persist, the validation of PPG applications in real-world conditions highlights their role in advancing accessible, scalable cardiovascular monitoring solutions^109^.

#### AI for ECGs acquired on wearable and portable devices

A major application of AI is wearable and portable device-acquired ECGs^110^. This is built on the foundation of developments in deep learning for ECGs that initially leveraged the 12-lead ECG data captured in clinical settings to detect signatures of structural and functional cardiovascular disorders. For example, neural networks applied to 12-lead ECGs demonstrated the ability to identify subtle signatures of range of structural heart diseases with high accuracy^111,112^.

Wearable and portable ECG devices have expanded the accessibility of AI-ECG-based cardiac screening. Our group developed a noise-adapted AI model on augmented single-lead ECGs that demonstrated resilience to real-world signal artifacts, identifying LV systolic dysfunction with an area under the receiver operating characteristic curve (AUROC) of 0.87 on noisy 1-lead ECG measurable on wearable devices^52^. Another study adopted a 12-lead model and achieved an AUROC of 0.88 for the same endpoint on Apple Watch ECGs. The study also highlighted the scalability of AI-enabled wearables by enrolling over 2400 participants remotely across multiple states, demonstrating high engagement and feasibility for population-level cardiac diagnostics^113^.

Beyond detecting systolic dysfunction, AI-ECG models have been applied to various other cardiovascular applications, including the detection of occult arrhythmias, predicting sleep apnea events, and screening for a broad range of structural heart disorders, such as functional, valvular, and infiltrative cardiomyopathies.

#### AI for handheld devices available in the community

POCUS is an accessible imaging modality increasingly integrated into clinical practice^114^. While it doesn’t necessitate extensive resources or advanced technology, acquiring high-quality ultrasound images requires training and experience^115^. AI-guided acquisition systems address this challenge by providing real-time feedback to users during scanning^115,116^. These systems analyze live images and offer guidance on probe positioning and movement, ensuring clinicians with limited ultrasound experience can capture images that can be more^116^. Beyond acquisition, AI tools have been developed to interpret POCUS images through advanced models capable of identifying clinical features typically inferred by experts reviewing high-quality imaging data^117^. Recent deep learning frameworks have demonstrated the ability to infer a full range of conditions from cardiac ultrasound even with limited imaging protocols, making it particularly valuable in resource-constrained and point-of-care acquisitions^118^. Recent work has also enabled the detection of a range of hidden signatures of disease from simple cardiac ultrasound^16^, detecting several underdiagnosed cardiomyopathies opportunistically, such as amyloid and hypertrophic cardiomyopathy, by being able to diagnose these conditions directly on real-world POCUS studies done in emergency departments, despite the low quality of these images^16^. The approach that integrated synthetic data augmentation to overcome the inherent variability of POCUS imaging highlights the role of carefully designed deep learning-enabled algorithms in reducing the burden of expert review in triaging care and inferring clinical information from this high-fidelity portable technology.

### Novel care paradigms powered by AI-enabled digital health devices

The current and emerging applications of AI to wearable and portable devices can enable broad changes to clinical care paradigms. Herein, we propose the applications.

#### Novel disease detection

The presence of low-cost wearable and portable devices directly in the communities unlocks the potential of scalability and accessibility for CVD screening. Most of these would represent the applications of AI to PPG and/or ECG as a first-level screen. However, given the low prevalence of many cardiovascular conditions in the community, the degree of false positives would overwhelm confirmatory testing systems. To address this, innovative detection systems tailored to low-prevalence settings are essential. These systems must incorporate strategic approaches within community-based disease detection programs. First, the technologies used must demonstrate consistent performance across broad demographic populations and those at risk for disease. Second, the deployment would have to be among a selected population with a certain threshold of prevalence. Third, whenever possible, a second-order screen would be done directly in the community, thereby reducing the number of referrals for confirmatory testing. An example of such an approach is the NIH-funded DETECT-AS study that is leveraging these three elements together in a multicenter randomized clinical trial^119^. It is a novel AS screen strategy that is deploying 1-lead portable ECG among older adults who have a higher prevalence of AS, with the model explicitly adapted to noisy acquisition on a portable device^119^. However, with an expected prevalence of 11% even among this selected population, 29% would be false positives. To address that, the study proposes to sequentially deploy an AI-assisted assessment of a novice-acquired cardiac POCUS to further enrich the screen, with the number of false positives expected to be 5%. While the performance of such tools in real-world practice remains to be established, the role of AI-assisted disease detection requires thoughtful design and explicit evaluation.

#### Cardiovascular risk prediction

AI-driven models are also increasingly being applied to wearable and point-of-care data to enhance cardiovascular risk prediction and personalized risk stratification. Unlike traditional risk scores, which often rely on a narrow set of variables, use static data points, and are typically validated only for specific populations with defined characteristics, AI-driven risk prediction models can leverage diverse and complex data sources, including real-time inputs from wearables, to stratify risk for a broader range of conditions. For instance, while traditional risk scores have focused on broad categories of conditions such as atherosclerotic cardiovascular disease and HF^22,23^, emerging AI-driven models can provide risk assessment on a range of individual structural, functional, and arrhythmic cardiac disorders. These models can also dynamically adjust to the unique risk profiles of individuals, enabling more inclusive and accurate predictions across diverse demographic and clinical populations.

For example, AI algorithms applied to wearable ECGs have proven effective in predicting the risk of new-onset HF. Positively screened individuals were found to have a 3- to 7-fold higher risk of HF hospitalization across diverse cohorts in the United States, United Kingdom, and Brazil^120^. These AI-based models significantly outperformed traditional risk scores. Additionally, AI-ECG applications for wearable data have been successfully used to predict SHDs, with up to a six-fold increased risk observed in individuals likely to develop SHDs^54^. Therefore, these community-centered and precise risk assessments can enable more targeted and efficient decision-making but without burdening systems with extensive evaluations.

#### Therapeutic monitoring

AI-powered applications for wearable data are transforming therapeutic monitoring, offering innovative tools to evaluate treatment response and track disease progression in cardiovascular care. In contrast to traditional monitoring approaches, which depend on periodic clinic visits and place a significant burden on the patient to engage with their care, AI-driven solutions enable continuous, real-time monitoring that can be delivered remotely directly in the community. These systems integrate data from multiple sensors and metrics combining these parameters to reduce the reliance on proactive patient involvement while delivering high-quality insights. For example, for HF monitoring, changes in activity and sleep can serve as early warning signs of a decompensation event^7,19^. The timely availability of this data empowers clinicians to deliver timely interventions, potentially improving the overall management of these patients. One additional example is wearable-collected ECGs to monitor real-world outcomes of therapies. A recent series of studies demonstrated AI-ECG can also be used to evaluate responses to therapies for diseases with complex underlying pathophysiological processes, such as HCM, identifying those with response to therapy with mavacamten, a targeted therapy compared with surgical management that focuses only on resecting the septum^87^. Therefore, as more disease-specific AI models are developed, it is likely that many unique biomarkers of treatment and recovery will emerge and potentially meaningfully change how we deliver care, especially through their extension to widely available portable and wearable devices.

#### Triaging of care for limited healthcare resources

Another key application that does not flow as an extension of the clinical use of these models is how they would enable the efficient allocation of limited public healthcare resources. Among individuals referred for advanced cardiac imaging, patients with the disease might experience significant delays in confirmatory testing and treatment, while others without the disease are demonstrated to have normal results. While this is expected on any diagnostic test, when a timely diagnosis can change disease trajectory, prioritization based on expected results can enable fairer resource allocation. In this context, AI applications leveraging wearable-collected data present a promising solution by facilitating risk-based prioritization of advanced diagnostics and care by a test that can be provided in the community. For example, AI-ECG-based risk predictions help prioritize patients for advanced imaging by predicting the likelihood of a series of cardiomyopathies^54,121^. Similarly, where clinical imaging is challenging to obtain but advanced phenotyping is needed beyond what is offered by AI-ECG, a community-facing POCUS device that is interpreted with automated tools^16,118^, can further offer an even stronger clinical rationale for triaging testing.

## Future directions

While wearable and portable devices powered by AI tools hold immense potential to transform community-based cardiovascular care, there are key proximate priorities: data standardization, equitable access with a participant-focused design, and robust ethical frameworks to regulate these technologies. To ensure scalability and equity, broader efforts are needed to address the digital divide that may limit access to AI-enabled wearables. Without these investments, there is a risk that such technologies may primarily benefit higher-income or tech-savvy populations, rather than closing the care gap they aim to address. The landscape of community and participant-powered networks is likely to mature and evolve with both opportunities for growth and additional challenges. It is imperative that these networks are nurtured with purposeful design, given their potential impact on resource-efficient and broadly accessible cardiovascular care. Future progress also depends on generating high-quality evidence through well-designed RCTs and prospective observational studies that demonstrate clinical utility and safety. In parallel, translating this evidence into practice will require sustainable reimbursement frameworks to support adoption within care models that prioritize value-based care. Moreover, to ensure these innovations are implemented responsibly and equitably across borders, global collaboration will be essential to advance international standards for data sharing, validation, and oversight, representing the foundation for trustworthy, accessible, and scalable cardiovascular care powered by AI-enabled community-based digital health devices. Table 1 summarizes the key challenges of incorporating AI-enabled consumer devices in cardiovascular care and potential solutions.

**Table 1:** Key challenges in the use of digital health devices for cardiovascular care and corresponding AI-driven solutions

Key challenge	AI-driven solution or strategy
Lack of data standardization across devices	Adoption of standards like HL7 FHIR or standardized ontologies
Data interoperability issues	AI-enabled harmonization using NLP, embedding models, and ontological mapping
Variable data quality and noise	Noise-adapted deep learning models and real-time signal quality feedback
Limited clinical context for wearable data	Linkage of wearable data with EHRs and structured clinical outcomes
Alert fatigue and workflow disruption	AI-generated prioritization algorithms and clinician-facing dashboards
Regulatory frameworks	Adaptive regulatory frameworks (e.g., FDA SaMD) with clearly defined use cases
Bias and equity concerns	Inclusion of diverse populations in the development and validation of models
Reimbursement and economic sustainability	Evidence-based cost-effectiveness analyses and integration with value-based care models
Patient adherence and engagement	Human-centered design with feedback loops and passive sensing to reduce user burden
Lack of data standardization across devices	Adoption of standards like HL7 FHIR or standardized ontologies

## Conclusion

AI-enhanced wearable and portable devices represent a transformative force in cardiovascular care by enabling efficient, equitable, and accessible care directly in the communities. This paradigm represents a shift from reliance on resource-intensive, expert-driven processes toward scalable, technology-driven solutions that improve the cardiovascular health and outcomes of our communities without overwhelming our limited healthcare resources. However, this utopic future of community-focused cardiovascular care requires the purposeful design and robust evaluation of novel care pathways that leverage these tools with a specific focus on accurate, equitable, and accessible care.

## Data Availability

No datasets were generated or analyzed during the current study.
